# Synergisms, Discrepancies and Interactions between Hydrogen Sulfide and Carbon Monoxide in the Gastrointestinal and Digestive System Physiology, Pathophysiology and Pharmacology

**DOI:** 10.3390/biom10030445

**Published:** 2020-03-13

**Authors:** Urszula Głowacka, Tomasz Brzozowski, Marcin Magierowski

**Affiliations:** Department of Physiology, Jagiellonian University Medical College, 16 Grzegórzecka Street, 31-531 Cracow, Poland; urszula.glowacka@uj.edu.pl (U.G.); mpbrzozo@cyf-kr.edu.pl (T.B.)

**Keywords:** hydrogen sulfide, carbon monoxide, heme oxygenase, cystathionine-γ-lyase, cystathionine-β-synthase, 3-mercaptopyruvate sulfur transferase, digestive system

## Abstract

Endogenous gas transmitters, hydrogen sulfide (H_2_S), carbon monoxide (CO) and nitric oxide (NO) are important signaling molecules known to exert multiple biological functions. In recent years, the role of H_2_S, CO and NO in regulation of cardiovascular, neuronal and digestive systems physiology and pathophysiology has been emphasized. Possible link between these gaseous mediators and multiple diseases as well as potential therapeutic applications has attracted great attention from biomedical scientists working in many fields of biomedicine. Thus, various pharmacological tools with ability to release CO or H_2_S were developed and implemented in experimental animal in vivo and in vitro models of many disorders and preliminary human studies. This review was designed to review signaling functions, similarities, dissimilarities and a possible cross-talk between H_2_S and CO produced endogenously or released from chemical donors, with special emphasis on gastrointestinal digestive system pathologies prevention and treatment.

## 1. Introduction

Hydrogen sulfide (H_2_S) and carbon monoxide (CO), next to nitric oxide (NO) are the most recently studied endogenous gaseous mediators. Chemically, exogenous CO is a colorless and odorless gas lighter than air, while colorless H_2_S has characteristic smell of rotten eggs. Both gaseous molecules were assumed for many years to be toxic for human body and when generated in the mammalian tissues, they were considered only as a by-product of metabolic processes or, as in case of H_2_S, as the end product of anaerobic respiration due to bacteria capable of utilizing inorganic sulfur substrates [1,2]. However, based on scientific evidence, the second beneficial face of CO and H_2_S has been reported recently. It has been shown that these gaseous mediators, similarly to NO, play an important beneficial functions in the body among others including the regulation of homeostasis, vasorelaxation, the regulation of various enzymes activity and the modulation of the particular genes expression [1,2,3,4]. These small gaseous molecules have become the main target of many investigations in the context of various diseases including gastrointestinal (GI) pathologies. However, due to toxicity of inhaled CO or H_2_S, novel compounds were designed and developed with ability to release small amounts of these gaseous molecules and are widely used as pharmacological tools under experimental conditions [5]. Interestingly, it seems that together with NO, all three mediators form a kind of endogenous triad interacting with each other [6]. However, these aspects have not been investigated deeply so far. Thus, within this article we aimed to explain and highlight possible mechanisms of interaction and the cross-talk between CO and H_2_S biosynthesis pathways, with special emphasis on the development, prevention and treatment of gastrointestinal (GI) and digestive system pathologies.

## 2. Overview on CO and H_2_S Physiology and Pharmacology in Digestive System

### 2.1. H_2_S Physiology and Pharmacology

H_2_S is known to modulate various biological functions on molecular, biochemical and even functional level under physiological and pathological conditions. This molecule can be formed in both, enzymatic and non-enzymatic processes. Three enzymes are involved in the enzymatic production of H_2_S in the body: cystathionine-γ-lyase (CTH), cystathionine β-synthase (CBS) and 3-mercaptopyruvate sulfurtransferase (MPST) [7]. CTH and CBS are located in cytosol and their activity requires pyridoxal-5’-phosphate (P5P; vitamin B6) as a cofactor. MPST is present within cytosol and mitochondria and is P5P-independent but works in co-activity with cysteine aminotransferase (CAT), which is necessary to convert cysteine to 3-mercaptopyruvate, a substrate for MPST [7]. As mentioned before, the colonic sulfate-reducing bacteria could be also the source of H_2_S in the GI tract [8]. The molecular targets of endogenous H_2_S include many physiologically important proteins, signaling targets including kinases, phosphatases, thiols, polysulfide’s, thiosulfate/sulfite, iron-sulfur cluster proteins, the anti-oxidant compounds and transcriptional factors affecting multiple cellular and molecular responses. Thus, this mediator is involved in the regulation of signaling pathways and genes expression [9].

Numerous studies have shown that H_2_S has anti-oxidative, anti-inflammatory and cytoprotective properties [5,10]. The anti-inflammatory effect of H_2_S is assumed to result in inhibition of endothelial leukocyte adhesion, modulation of inflammatory markers expression, prostaglandins biosynthesis and by interacting with transcription factors such as nuclear factor kappa-light-chain-enhancer of activated B cells (NF-κB) and nuclear factor erythroid 2-related factor 2 (Nrf-2) [10,11]. NF-κB regulates the expression of genes encoding pro-inflammatory cytokines, some growth factors, cyclooxygenase-2 (COX-2) and apoptotic pathways components [12]. Its activation has been noted in various inflammatory diseases, such as rheumatoid arthritis, multiple sclerosis, atherosclerosis, systemic lupus erythematosus, type I diabetes, chronic obstructive pulmonary disease and asthma, and especially in digestive system pathologies, including e.g., inflammatory bowel disease (IBD mainly consisting of Crohn’s disease and ulcerative colitis) and drugs-induced gastrotoxicity [13,14,15]. NF-κB inhibition by H_2_S can occur in two possible ways, by sulfhydration of the free thiol group on 38 cysteine in the subunit 65 or due to phosphorylation suppression and degradation of IkBα [16,17]. As it has been mentioned above, H_2_S can also interact with another transcription factor, Nrf-2, which regulates cellular defensive response to inflammation and oxidation [11,18]. Physiologically, Nfr-2 is present in the cytoplasm as a molecular complex with the cytoskeleton protein, Keap1. When cellular redox homeostasis is imbalanced, Nrf-2 is released from its repressor (Keap1), translocated to the nucleus and bound to promoter of antioxidant responsive element (ARE). This translocation leads to increased transcription for anti-oxidative genes, such as catalase (CAT) or superoxide dismutase (SOD) [19,20], glutathione-S-transferase (GST) and glutathione peroxidase (GPx) [11,18]. H_2_S most likely activates Nrf-2 pathway by sulfuration the Keap1 protein (at the cysteine-151, 226 and 613), leading to this nuclear factor release, its nuclear translocation and enhancement of antioxidant genes expression [18]. H_2_S is also supposed to exert its anti-inflammatory activity due to the modulation of annexin A1 (AnxA1) pathway. H_2_S triggers AnxA1 mobilization and modulates vascular inflammatory processes by the enhancement in the detachment rate of leukocytes from the vessel wall resulting in reduction of inflammation [21]. This gaseous molecule can interact with the endogenous NO in vasomotor responses of the arterial vessels involved in the blood pressure control. Previous study concerning the systolic blood pressure (sBP), vasoreactivity, NO-synthase (NOS) expression and activity, CTH expression and geometry of the isolated thoracic aorta revealed that this vasomotor interaction between H_2_S released from Na_2_S with NO in thoracic aorta of normotensive and spontaneously hypertensive rats (SHRs) seems to be age- and blood pressure-dependent [4]. 

It has been reported that H_2_S biosynthesis pathway activity could be crucial for gastric mucosal barrier physiology and alterations in this gaseous molecule signaling were observed in pathology of upper GI-tract and other parts of the digestive system [5]. Moreover, H_2_S-releasing NaHS has been shown to accelerate ulcer healing and to attenuate non-steroidal anti-inflammatory drugs (NSAIDs)-induced gastrotoxicity [22,23]. Interestingly, H_2_S-releasing derivative of naproxen (ATB-346) which successfully completed phase 2 clinical trial, offers a promising safer alternative for conventional native form of this NSAID not only in improvement of pain relief but also in GI-safety manifested by the reduction of upper GI tract damage formation in subjects treated with equimolar effective doses of ATB-346 versus naproxen [24]. Similarly GYY4137, slow H_2_S-releasing compound has been shown to exert anti-inflammatory activity [25] and to decrease the area of ischemia/reperfusion-induced gastric damage in rats [23]. GYY4137 also attenuated intestinal barrier injury in mouse model as well as in human colon cancer cells model of lipopolysaccharide (LPS) or TNF-α/IFN-γ-induced endotoxemia [26].

It is also worth mentioning that natural sources of sulfur compounds, such as garlic-derived compounds including diallyl sulfide (DAS) disulfide, diallyl disulfide (DADS), diallyl trisulfide (DATS) and allicin were described as H_2_S releasing molecules [27]. Numerous studies have shown beneficial therapeutic and protective effects of these compounds in cancer development [24,25,26]. Cell cultures and animal models, as well as epidemiology data have revealed the chemopreventive activity of these H_2_S donors, especially in gastric and colorectal cancers [28,29,30]. On the other hand, somehow related to H_2_S and sulfide physiology, sulfiredoxin (Srx) is multifunction enzyme involved in antioxidant metabolism by reduction of cysteine sulfinic acid to sulfenic acid in proteins exposed to oxidative stress [31]. Some studies suggested that increased Srx expression could be linked with carcinogenesis and tumor progression [29,31]. Indeed, in gastric tumors cells, expression of Srx was significantly elevated as compared with normal tissue [29]. Interestingly, it has been reported that treatment with DATS decreased expression of Srx in gastric tumor cell line BGC823 [29]. Similarly, Srx is highly expressed in poorly differentiated, aggressive HCT116 human colorectal cancer cells, while in normal colon epithelium (NCM460) or cells derived from well-differentiated colorectal carcinomas (SW640 and HT29) this protein was not detected [32]. It has been also observed that inhibition of Srx resulted in selective death of cancer cells by disturbance in redox homeostasis [33]. Taken together, we assume that inhibition of Srx could be considered as a novel approach and target for anticancer treatment. On the other hand, some studies showed pro-cancer effects of H_2_S [34,35,36]. This phenomenon may be associated with induction of angiogenesis, regulation of mitochondrial bioenergetics, acceleration of cell cycle progression, and anti-apoptotic actions [34]. It has been reported that in adenocarcinoma-derived cell lines (HCT-116, HT-29, LoVo) CBS expression was upregulated as compared to control-non-malignant colonic epithelial cells [35]. Moreover, anti-apoptotic effect of H_2_S and its involvement in the enhancement of cell proliferation linked with alterations in CTH expression was demonstrated on  human gastric adenocarcinoma (AGS) [36]. Taken together, H_2_S has been shown to exert both pro- and anti-cancer effects and we assume that this could be dependent on the dose of this gaseous mediator and possibly on the differences in sensitivity of various cell types to the impact of this molecule.

Garlic-derived H_2_S-releasing compounds were also shown to exhibit hepatoprotective effect in experimental animal models [37,38]. Pretreatment with DAS decreased NF-κB and TNF-α in serum and reversed the decreased level of superoxide dismutase (SOD) and catalase activity in liver as observed in a well described model of liver injury induced by carbon tetrachloride (CCl_4_) [38]. Therefore, garlic-derived H_2_S donors might affect cell signaling networks in a similar way as synthetic H_2_S donors.

### 2.2. CO Physiology and Pharmacology 

CO is produced in mammalian tissues by heme degradation involving enzymatic activity of heme oxygenases (HMOXs) [2]. This protein has two main isoforms, HMOX-1 and HMOX-2 translated by the expression of two separate genes. HMOX-1 is inducible and this isoform is active under imbalanced homeostasis conditions, while HMOX-2 is constitutively expressed with relatively low yield. Interestingly, rats also encode third isoform, HMOX-3, probably from a pseudogene that does not produce a functional form of this protein [39]. It has been indicated that in majority of biological systems, CO has been shown to exert anti-inflammatory, anti-proliferative, anti-apoptotic and cytoprotective effects, similarly to H_2_S [39,40]. CO may regulate the activity and functionality of various proteins by binding to heme domains. This includes e.g., hemoglobin, myoglobin, cytochrome c, cytochrome P450, nitric oxide synthase (NOS), catalase, prostaglandin H synthase, nicotinamide adenine dinucleotide phosphate (NADPH) oxidase (Nox) and transcription factors NPAS2, Bach-1 and Bach-2 [41]. 

Recently, endogenous CO or that released from the synthetic CO donor, tricarbonyldichlororuthenium (II) dimer (CORM-2) has been implicated in the mechanism of gastric mucosal integrity, gastroprotection and the healing of chronic gastric ulcers [42,43]. These gastroprotective and therapeutic effects of CO are mediated by the activation of soluble guanylyl cyclase (sGC), which is regulating cyclic guanosine monophosphate (cGMP) generation. Elevated intracellular concentration of this second messenger leads to enhancement in the gastric microcirculation as documented by the direct measurement of gastric blood flow (GBF) [40,42,43]. Moreover, the potential mediators of this CO-induced beneficial action such as Ca^2+^-activated K^+^ channels [44] and the arachidonic acid derivative (20-HETE) production, have been proposed to mediate vasoactive, gastroprotective and ulcer healing properties of CO [45]. 

In addition, the anti-apoptotic and anti-hypoxic effects of this gaseous molecule have been demonstrated [37]. In animal in vivo and cell culture in vitro models, CO has been shown to inhibit LPS-induced overexpression of pro-inflammatory cytokines, such as TNF-α, IL-1β, IL-6, macrophage inflammatory protein (MIP)-1β and granulocyte-macrophage colony-stimulating factor (GM-CSF) [44]. It has been assumed that the anti-inflammatory activity of CO is related to the possible modulation by this gaseous molecule of the multiple members of the mitogen-activated protein kinase (MAPK) family [44]. Fukuda et al. has reported that water-soluble CORM-3 attenuated inflammatory response in animal model of trinitrobenzenesulfonic acid (TNBS)-induced colitis by targeting CD4+ T cells releasing pro-inflammatory cytokines (e.g., TNF-α, IL-8, INF-γ, IL-17A) [46]. Interestingly, CO may affect ERK MAPK pathway in T cells inhibiting their proliferation followed by the reduced pro-inflammatory cytokines release, as it has been documented in experimental mice model of colitis [47]. Moreover, recent evidence has indicated that pretreatment with CORM-3 alleviated the postoperative ileus (POI) in mice [48]. Similarly to above mentioned observations, CORM-3 significantly reduced intestinal inflammation and oxidative stress in postoperative ileus due to activation of p38 MAPK and downregulation of ERK1/2 [48]. Taken together, it can be assumed that CO has immunomodulatory properties and affects the activity of various cell types including T cells, B cells, epithelial cells, neutrophils, mast cells, dendritic cells and macrophages stimulated in the course of gastrointestinal digestive disorders [49]. Takagi et al. investigated the effects of CO-releasing CORM-A1 on Th17 differentiation using T-cell transfer-induced colitis in mice [50]. They revealed that CORM-A1 ameliorated intestinal inflammation through reduction of retinoid related orphan receptor (ROR)γ-receptor expression, inhibition of Th17 differentiation and by the decrease of IL-17A level [50]. HMOX-1/CO pathway can also regulate intestinal inflammation in acute and chronic experimental models by cross-talk of this pathway proteins with enteric microbiota in mucosal immune compartment [49]. Undoubtedly, CO could be considered as potential therapeutic agent in various GI disorders due to the wide range of molecular inflammatory and anti-inflammatory targets affected by this small gaseous molecule. 

## 3. Similarities and Dissimilarities in H_2_S and CO Activity and their Interaction within Digestive System

### 3.1. Parallelisms and Discrepancies in CO and H_2_S Effects and Targets

CO and H_2_S, likewise NO have similar activity and regulate parallel molecular pathways, also due to mutual interaction and the cross-talk between these molecules [51,52]. H_2_S and CO were shown to prevent gastric mucosa against NSAIDs- or alendronate-induced damage and to accelerate ulcer healing due to an improvement of gastric microcirculation [15,53,54,55]. Both molecules attenuated hypoxia and inflammation decreasing gastric mucosal expression of HIF-1α and NF-κB [15]. Recent evidence indicates that H_2_S- and CO-gastroprotection was abolished or at least reduced by the inhibition of sGC or NOS activity [54,56]. Interestingly, in contrast with H_2_S, CO was shown to maintain its gastroprotective effects independently on afferent sensory nerves activity [56]. On the other hand, H_2_S-releasing NaHS accelerated gastric ulcer healing but in contrast with CO-releasing CORM-2, this effect of H_2_S donor was accompanied by the upregulation of gastric mucosal protein expression for Nrf-2, vascular endothelial growth factor (VEGF) and epidermal growth factor receptor (EGFr) at the ulcer margin [42,53]. 

Interestingly, it has been reported that CO donors could exert antibacterial activity against *Helicobacter pylori* (*H. pylori*) [57]. Nowadays, the gastric colonization with *H. pylori* constitutes the major risk of gastric and duodenal ulcer diseases, mucosa-associated lymphoid tissue (MALT) lymphoma and even gastric adenocarcinoma [58]. Antimicrobial action of stimulated murine macrophages was enhanced by CORM-2 against *H. pylori* [57]. Moreover, CORM-2 impaired *H. pylori* respiration and inhibited *H. pylori* related urease activity [57], however, the role of H_2_S in *H. pylori* infection has not been fully recognized. On the one hand, *H. pylori* produces H_2_S [8], but on the other hand, as mentioned above, natural sulfur compounds like garlic have antibacterial activity [59]. Moreover, microbiological studies revealed anti-*H. pylori* potential of DADS derived from garlic powder or garlic oil [59]. Antibacterial activity of garlic-derived compounds were shown to be effective in patients infected with *H. pylori* [60]. Furthermore, allicin as an adjuvant to conventional anti-*H.pylori* therapy increased efficiency of *H. pylori* eradication [61]. However, further studies are required to fully explain these bactericidal aspects of H_2_S donating agents.

Both H_2_S and CO donors were shown to increase HCO_3_^-^ secretion in rat duodenum protecting the duodenal mucosa against the damage induced by acidic content [62]. Additionally, H_2_S was observed to modulate gastric secretion possibly via activation of TRPV1 channel and the consequent release of substance P and in a NF-κB -dependent manner [63]. H_2_S released from NaHS stimulated the secretion of HCO_3_^-^ in part mediated by the activity of capsaicin-sensitive afferent neurons as well as endogenous NO and PGs [64]. Similarly, CORM-2 dose-dependently elevated HCO_3_^-^ secretion acting as the stimulant of endogenous PGs biosynthesis [62]. 

In another study, de Araujo et al. proposed that adenosine monophosphate-activated protein kinase (AMPK) plays an important role as a regulator of cellular energy and metabolism, and could be the common target for all above mentioned gaseous mediators [65]. Indeed, AMPK inducers can actually exert a beneficial effects within the GI tract, e.g., metformin has been shown to suppress esophageal squamous cell carcinoma (ESCC) [66]. Interestingly, the γ-subunit of AMPK contains four CBS domains located close to the N-terminus of this subunit, operating in pairs known as Bateman’s domain [67]. The administration of H_2_S, CO and NO donors increased p-AMPK expression and protected gastric mucosa of mice against ethanol-induced lesions [65]. On the other hand, it has been also indicated that AMPK stimulates HMOX-1 gene expression within human vascular cells and rat arteries *via* modulation of Nrf2/ARE pathway [6].

**Figure 1:**
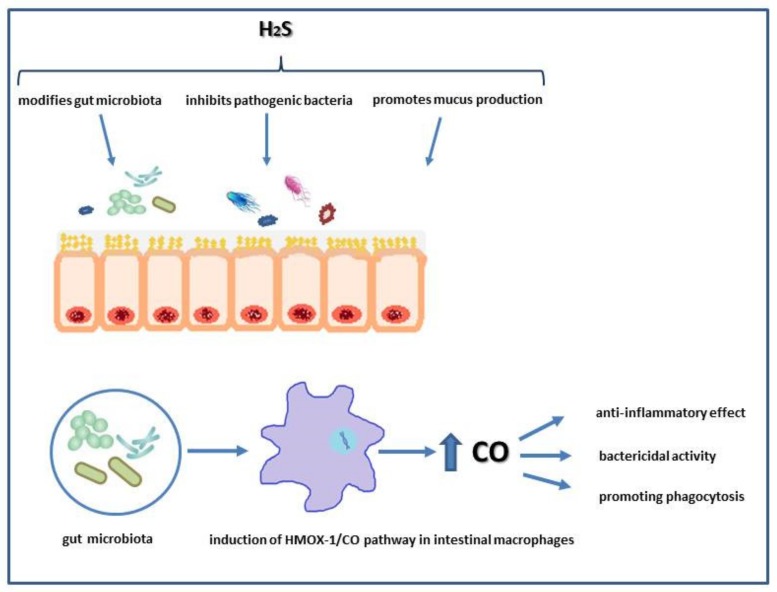
Possible involvement of hydrogen sulfide (H_2_S) and carbon monoxide (CO) in physiology of intestinal microbiota.

Interestingly, H_2_S donor, DADS has been demonstrated to stabilize hypoxia-inducible factor α (HIF-1α) and to prevent colonic mucosa in experimental model of colitis [68]. H_2_S also is produced by intestinal bacteria forming a biofilm lining the mucus surface [69]. Dysbiosis of the gut microbiota and “leaky” mucus layer is associated with the pathogenesis of IBD, irritable bowel syndrome (IBS), colorectal cancer and extra-intestinal disorders like obesity or metabolic syndrome [70,71]. It has been reported that H_2_S derived from DADS can have additional protective effect on gut affecting intestinal microbiota and biofilm formation because treatment with this compound not only alleviated intestinal damage but has also effectively reconstituted microbiota biofilm structure in rat model of colitis [69,72]. As mentioned before, CO due to its anti-inflammatory activity ameliorated intestinal injury in experimental models of colitis by the modulation of pro-inflammatory cytokine expression [49]. Experiments carried out in mice showed that enteric microbiota have the ability to regulate the activity of intestinal macrophages essential in killing pathogenic bacteria such as *Salmonella enterica*. This bactericidal effect was associated with an induction of HMOX-1/CO pathway by microbiota [49,73]. Moreover, the gut microbiota can produce CO due to the presence of enzymes with similar functions as HMOX-1. In addition, CO can directly interact with heme-containing groups in some intestinal bacteria [73]. Importantly, in patients with ulcerative colitis, increased intestinal expression of HMOX-1 has been demonstrated [74]. Thus, the interplay between gasotransmitters and gut microbiome may play an important role in maintenance of intestinal homeostasis (Figure 1).

### 3.2. Cross-talk between CO and H_2_S Biosynthesis Pathways

#### 3.2.1. Effects of CO on H_2_S Biosynthesis Pathway 

CO has the chemical ability to bind to metal-centered prosthetic groups of many proteins including enzymes [75]. Therefore, heme containing proteins could be considered as the main target of molecular events associated with CO activity. CBS, similarly to CTH and MPST takes part in endogenous H_2_S production but belongs to the above mentioned type of enzymes. Indeed, it has been reported that CO can bind to the prosthetic heme domain of CBS, stabilizing CO-Fe(II)-histidine complex and in turn, resulting in this enzyme inhibition [75,76]. CO regulates H_2_S production, but on the other hand, CBS acts in parallel as a CO sensor [75], however, the indirect effect of CO on CTH and MPST activity could not be excluded. 

**Figure 2:**
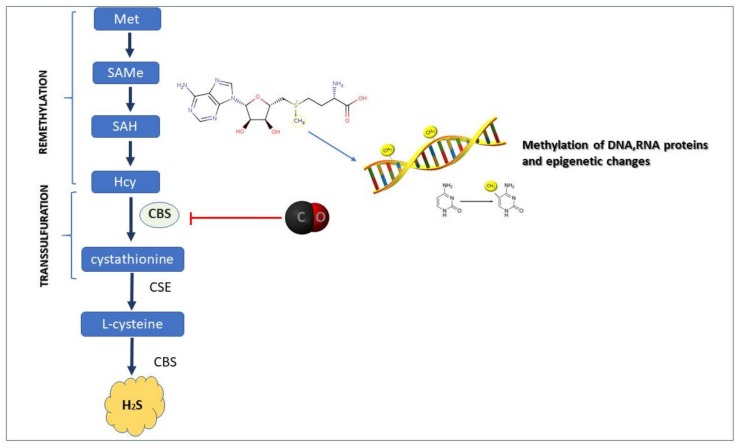
Possible impact of carbon monoxide (CO) on cystathionine-β-synthase (CBS) activity resulting in transsulfuration/remethylation switch. Abbreviations: Met: methionine; SAMe: S-adenosyl-methionine; SAH: S-adenosyl-homocysteine; Hcy: homocysteine; CSE: cystathionine-γ-lyase

With the implementation of metabolomic methods, the decreased activity of CBS, which affected remethylation and transsulfuration has been observed (Figure 2). In detail, the CBS inhibition by CO has been shown to elevate methionine and S-adenosylmethionine (SAMe) levels resulting in modulation of proteins methylation leading to increased generation of anti-oxidants [77,78]. Therefore, CO-mediated CBS inhibition is supposed to switch the transsulfuration pathway into the remethylation pathway, leading to the wide range of proteins and histones methylation. Indeed, based on in vitro model, it has been demonstrated that CO released from CORM-2 enhanced histone H3 protein methylation in human monoblastic leukemia U937 cells [79].

Histone modification and DNA methylation work together in mechanism of chromatin condensation process which is a morphological hallmark of apoptosis, and its regulatory availability for transcription factors. For instance, alterations in DNA methylation have been reported in development of both precancerous Barrett’s esophagus (BE) and esophageal adenocarcinoma (EAC), despite its noticeable role in gastric carcinogenesis [80]. Histological alterations that occur in BE include the transformation of normal squamous epithelium into intestinal metaplasia with a further progression into dysplasia and finally into adenocarcinoma. Thus, there were also epigenetic alterations observed, especially within DNA methylation. Genomic analysis revealed the presence of global hypomethylation during the course of metaplasia development [81]. However, the possible involvement of CO and H_2_S in the development of chronic esophageal disorders has not been fully elucidated. Taken together, this aspect should be further investigated with the emphasis on possible impact of these gaseous mediators in the mechanism of DNA methylation process of these upper GI pathologies.

Interestingly, the interaction of CO with CBS/H_2_S pathway could be involved in regulation of bile secretion since CO attenuated H_2_S levels, stimulated biliary HCO_3_^-^ and therefore protected the liver, accelerating its detoxification [82]. In fact, H_2_S may play an essential role in these processes because heterozygous knockout (CBS^+/-^) reversed these effects of CO [82]. However, the inhibition of CTH activity by propargylglycine (PAG) induced choleresis in the rat liver suggesting that the increased CO and decreased H_2_S content are responsible for an elevation of bile secretion [83]. Since bile secretion strictly depends on the blood flow in mesenteric circulation, the blood vessel vasomotor activity can be directly mediated by these endogenous gaseous molecules. Indeed, the anti-contractile effect of H_2_S in both, the rat vascular wall and perivascular tissue has recently been demonstrated using normal and SH rats [3,4]. It has been found that pretreatment with PAG elevated the noradrenaline-induced contraction in normal but not in spontaneously hypertensive (SH) rats [3]. However, perivascular adipose tissue presence increased vasoactive effect of exogenously derived H_2_S in SH rats [3]. 

On the other hand, an opposite final effect of CBS inhibition by CO, such as increased H_2_S concentration has been proposed [77]. This could be explained by accumulation of homocysteine that compensatively raised CTH activity in endoplasmic reticulum stress model in HEK293 cells, an effect also confirmed in CBS deficient mice [84]. This hypothesis could be corroborative with our previous finding that pretreatment with CO-donor, CORM-2 not only prevented gastric lesions induced by aspirin but also downregulated gastric mucosal protein expression of CBS and subsequent H_2_S production [54]. However, in chronic experimental gastric ulcer study, the daily treatment with CO donor throughout 9 days period, accelerated ulcer healing and upregulated mRNA expression for CTH and CBS increasing H_2_S production at the gastric mucosa of ulcer margin [53]. These observations strongly suggest that the effect of CO on CBS and possible activation of compensative mechanisms regulating H_2_S biosynthesis could be time-dependent.

#### 3.2.2. Effects of H_2_S on CO Biosynthesis Pathway

**Figure 3:**
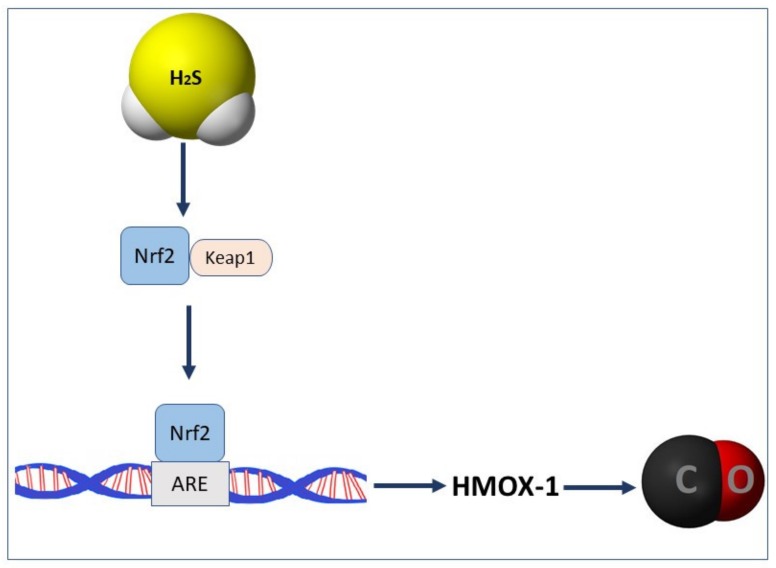
Nuclear factor erythroid2-related factor 2 (Nrf-2) mediated modulation of carbon monoxide (CO) production by hydrogen sulfide (H_2_S). Abbreviations: Keap1: Kelch-like ECH-associated protein 1; ARE: antioxidant response element

As mentioned above, the endogenous synthesis of CO depends upon the activity of HMOX-1 and HMOX-2. As reported previously, H_2_S can affect the expression of HMOX-1 and endogenous CO content indirectly, by modulation of Keap1-Nrf2 pathway [58] (Figure 3). For instance, the pretreatment with H_2_S-releasing derivative of naproxen, ATB-346 increased the expression of Nrf-2 and HMOX-1 proteins in gastric mucosa compromised by acute stress [32].

As reported by our group previously, daily treatment with H_2_S-releasing NaHS accelerated gastric ulcer healing and decreased mRNA expression of HMOX-1 at ulcer margin supporting the notion that this beneficial effect of H_2_S donor is mediated by anti-inflammatory activity of this gaseous molecule [53]. However, pharmacological inhibition of HMOXs by administration of zinc protoporphyrin IX (ZnPP) reversed gastroprotective, ulcer healing and vasodilatory effects of H_2_S donor [53,54]. Interestingly, beneficial outcomes of CORM-2 administration in gastric mucosa and gastric microcirculation were still observed despite the H_2_S biosynthesis pathway has been pharmacologically inhibited [43].Taken together, these results suggest that, at least within gastric mucosa, H_2_S activity including modulation of gastric microcirculation is dependent on endogenous CO biosynthesis while beneficial effects of CO are independent on the activity of endogenous H_2_S biosynthesis pathway. 

## 4. Conclusions, Possible Implementation into Therapy of GI Disorders and Future Perspectives 

**Figure 4:**
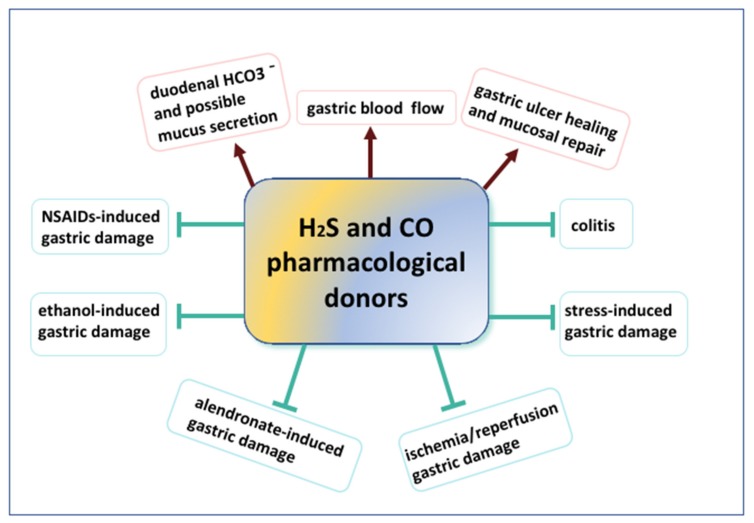
Schematic overview of beneficial actions of hydrogen sulfide (H_2_S) or carbon monoxide (CO) releasing pharmacological tools in physiology and pathophysiology of digestive system pathologies.

CO and H_2_S donors were shown to exert preventive and therapeutic effects in various digestive system disorders and pathologies, such as drugs-induced gastrotoxicity, ulcer healing or prevention and treatment of colitis (Figure 4). It has been reported that these gaseous molecules have similar molecular targets and could influence each other (Table 1). Nevertheless, the emerging cross-talk and interactions between these two molecules remain to be studied. Especially, possible effects of both gaseous molecules and novel class of drugs releasing H_2_S and CO in the therapy of the short-term and long-term esophageal pathologies such as GERD, BE or EAC should be further determined. Additionally, influence of CO and H_2_S on the *H. pylori* infection consequences requires further investigations. Lastly, but not limited to, precise mechanisms and effects of CO and H_2_S on the methylation process and regulation of mitochondrial activity, especially in the context of upper GI pathologies could significantly expand the current knowledge related to the possible molecular targets of these gaseous transmitters and pharmacological agents releasing these gaseous molecules.

Even though the experimental evidence on protective efficacy of CO-donating agents are promising, clinical implementation of these compounds into therapy in humans might be questionable since many CO-releasing pharmacological tools contain heavy metals in their structure [85]. However, novel CO-releasing prodrugs were developed recently and further studies will hopefully reveal their clinical potential [86]. It is worth to highlight, that H_2_S-releasing derivative of naproxen, ATB-346 with its reduced gastrotoxicity passed successfully the Phase 2 of clinical trial [24]. Taken together, there are still many missing aspects to be answered and extensively investigated in the context of CO and H_2_S-related physiology and pharmacology of GI digestive system.

## Figures and Tables

**Table 1:** Summary of exemplary beneficial effects of H_2_S and CO.

	H_2_S 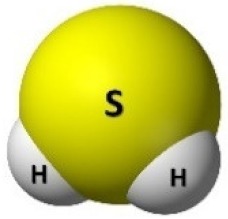	Reference	CO 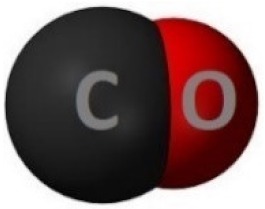	Reference
Beneficial effects of H_2_S and CO				
Anti-inflammatory	decreased serum level of TNF-α and IL-1β and expression of mRNA in gastric mucosa	[5,15]	inhibited production of TNF-α, IL-1β in LPS-stimulated macrophages in vivo and in vitro	[44]
	reduced mRNA and protein expression of HIF-1α in gastric mucosa	[5,15]	increased IL-10 expression in macrophages via activation p38MAPK	[87]
	supressed NF-κB pathway in gastric mucosa	[21]	decreased ERK1/2 kinase activity in T cells	[88]
	induced activation of AnxA1 pathway	[21]	re reduced mRNA and protein expression of HIF-1α in gastric mucosa and supressed NF-κB pathway in gastric mucosa	[15]
			involved in regulation of Th1, Th2, and Th17 lymphocyte differentiation, decrease of IL-17A content	[50]
Anti-oxidative	caused Nrf-2 /HMOX-1pathway upregulation	[11,18]	inhibited the lipid peroxidation	[2]
	decreased level of MDA and increased production of glutathione (GSH)	[7,56]	decreased level of MDA and modulated SOD activity	[56,89]
Vasodilatation	increased gastric microcirculation via sGC on endogenous NO and CO biosynthesis-dependent manner	[53,54,56]	Increased gastric microcirculation via sGC with contribution of NO biosynthesis pathway and independently on endogenous H_2_S activity	[40,42,43,54,56]
	dependent on activation of K_ATP_ channels	[90]	dependent on activation of K_ATP_ channels	[91]
HCO_3_^-^ secretion in duodenum	increased	[64]	increased	[62,64]
Impact on gut microbiota	caused the reconstitution of microbiota biofilm dysbiosis	[69,72]	found to be involved in CO/HMOX-1 pathway in cross-talk between the microbiota and the mucosal immune compartment	[49]
**Cross-talk between H_2_S and CO**				
**Direction**	**Mechanism of action**	**Possible biological effect**		**References**
CO→ ↓ H_2_S	CO can bind to CBS and inhibits its activity	switch of transsulfuration pathway into the remethylation pathway→ methylation of proteins→ epigenetic changes		[76,77]
H_2_S → ↑CO	H_2_S activates Nrf-2 which and modulates of HMOX-1 expression and CO production	modulation of oxidative homeostasis and Nrf-2-dependent molecular pathways		[18]
